# PlantPAN: Plant promoter analysis navigator, for identifying combinatorial *cis*-regulatory elements with distance constraint in plant gene groups

**DOI:** 10.1186/1471-2164-9-561

**Published:** 2008-11-26

**Authors:** Wen-Chi Chang, Tzong-Yi Lee, Hsien-Da Huang, His-Yuan Huang, Rong-Long Pan

**Affiliations:** 1 Institute of Bioinformatics and Structural Biology, National Tsing Hua University, Hsin-Chu 300, Taiwan; 2 Institute of Bioinformatics, National Chiao Tung University, Hsin-Chu 300, Taiwan; 3 Department of Biological Science and Technology, National Chiao Tung University, Hsin-Chu 300, Taiwan; 4 College of Life Sciences, National Tsing Hua University, Hsin-Chu 300, Taiwan

## Abstract

**Background:**

The elucidation of transcriptional regulation in plant genes is important area of research for plant scientists, following the mapping of various plant genomes, such as *A. thaliana*, *O. sativa *and *Z. mays*. A variety of bioinformatic servers or databases of plant promoters have been established, although most have been focused only on annotating transcription factor binding sites in a single gene and have neglected some important regulatory elements (tandem repeats and CpG/CpNpG islands) in promoter regions. Additionally, the combinatorial interaction of transcription factors (TFs) is important in regulating the gene group that is associated with the same expression pattern. Therefore, a tool for detecting the co-regulation of transcription factors in a group of gene promoters is required.

**Results:**

This study develops a database-assisted system, PlantPAN (Plant Promoter Analysis Navigator), for recognizing combinatorial *cis*-regulatory elements with a distance constraint in sets of plant genes. The system collects the plant transcription factor binding profiles from PLACE, TRANSFAC (public release 7.0), AGRIS, and JASPER databases and allows users to input a group of gene IDs or promoter sequences, enabling the co-occurrence of combinatorial transcription factor binding sites (TFBSs) within a defined distance (20 bp to 200 bp) to be identified. Furthermore, the new resource enables other regulatory features in a plant promoter, such as CpG/CpNpG islands and tandem repeats, to be displayed. The regulatory elements in the conserved regions of the promoters across homologous genes are detected and presented.

**Conclusion:**

In addition to providing a user-friendly input/output interface, PlantPAN has numerous advantages in the analysis of a plant promoter. Several case studies have established the effectiveness of PlantPAN. This novel analytical resource is now freely available at .

## Background

The appropriate regulation of gene expression is essential for all cellular processes, in which transcriptional control is primarily concerned with improved survival. In animals and plants, transcription factors are key regulators of gene expression and play a critical role in the life cycle [1]. Investigations on transcription factors (TFs) and their corresponding *cis*-acting elements in promoters have attracted much attention from researchers of gene regulation. However, defining all functional binding sites within an identified promoter is difficult, and the existence of some additional binding sites should be assumed [2]. Furthermore, studies of various model systems have shown that relatively few transcription factors can establish strikingly complex spatial and temporal patterns of gene expression [3]. Some co-regulatory networks model all significant associations among transcription factors in regulating common target genes [4]. Accordingly, work on the combinatorial interaction of transcription factors (TFs) is important in gene regulation. In a previous study, AthaMap [5,6] identified the co-localization of transcription factor binding sites and noted that the analysis of gene co-expression is crucial to reconstructing gene regulatory networks for plant scientists. The PathoPlant [7] web tool enables identification of plant genes co-regulated in plant defense response. Subsequently, common *cis*-regulatory elements in co-regulated genes are identified by exporting sets of genes to AthaMap. The study describes an effective resource, PlantPAN (Plant Promoter Analysis Navigator), for identifying the co-occurrence of transcription factor binding sites (TFBSs) in a group of gene promoters with distance constraint between two TFBSs, and presents graphically the transcription factor binding sites in specific gene promoter regions of interest. With the advent of microarray technology, *Arabidopsis *co-expression tool (ACT) [8] was developed as a tool for analyzing co-expression patterns across selected genes. ATTED-II [9] provides co-regulated gene relationships based on co-expressed genes deduced from microarray data and predicted *cis*-regulatory elements in the 200 bp region upstream of the transcription start site. Recently, Chawade *et al*. proposed putative cold acclimation networks by combining data from microarrays, promoter sequences and known promoter binding sites [10]. Accordingly, the "Gene Group Analysis" function in PlantPAN is useful for discovering co-regulated TFBSs in sets of plant genes and not restricted to a set of co-expressed genes of microarray data.

Many databases harbor collections of numerous transcription factors and are useful for the prediction of transcription factor binding sites in the promoter regions of plants. For instance, TRANSFAC [11-13] is a database of transcription factors, including genomic binding sites and DNA-binding profiles. Athena [14] is a database, which contains 30,067 predicted *Arabidopsis *promoter sequences and consensus sequences for 105 previously characterized transcription factor binding sites (TFBSs) and provides analysis on over-represented TFBSs occurring in multiple promoters. PlnTFDB [15] is an integrative plant transcription factor database that provides a web interface to access large (close to complete) sets of transcription factors of several plant species. PLACE [16] is a database that collects various *cis*- and *trans*- acting regulatory DNA elements, described in earlier studies[16]. AGRIS [17] contains an *Arabidopsis thaliana *transcription factor database (*At*TFDB) consisting of approximately 1,770 *Arabidopsis *TFs and their sequences (protein and DNA) grouped into around 50 families with information on available mutants in the corresponding genes. AGRIS [17] integrates a variety of tools to determine transcription factors and their putative binding sites on all genes to reconstruct transcriptional regulatory networks in *Arabidopsis*. JASPAR [18,19] is an open-access database of annotated, high-quality, matrix-based transcription factor binding site profiles for multicellular eukaryotes. DATF [20] stores information on 3D structural templates, EST expression, transcription factor binding sites and nuclear location signals (NLSs) of known and predicted *Arabidopsis *transcription factors. PlantCARE [21] is a database of plant *cis*-acting regulatory elements and a portal to tools for the *in silico *analysis of promoter sequences. AthaMap [5] contains 103 transcription factors and nearly 10 million putative TFs binding sites mapping *cis*-regulatory elements in *Arabidopsis*. Notwithstanding the recent development of the above resources, advances in plant science require a more detailed analysis of plant promoters. For example, CpG islands in the genome are important because of their strong correlation with gene regulation. CpG-rich regions are methylated and are associated with inactive DNA often linked to heterochromatin, gene silencing, and pathogen control [22-25]. In plants, DNA methylation is not only found on the cytosine of CpG islands, but also on CpNpG islands and nonsymmetrical trinucleotides [26-28]. Therefore, methods for identifying CpG/CpNpG islands, which are important sites for DNA methylation that may result in gene silencing, are certainly crucial [26-28]. Recently, CpGProD [29] and CpG Island Searcher [30] were developed to identify CpG/CpNpG islands in promoters. Tandem repeats in promoters are also critical as they participate in gene expression regulation as well [31-33]. For instance, a tandem-repeat rsus3 promoter construct displays three fold higher expression level in a GUS reporter gene assay experiment in *Oryza sativa *[32]. Moreover, in *Arabidopsis*, gene expression is up-regulated when gene promoters were enriched in GGCCCAWW and AAACCCTA repeat sequence; gene expression is down regulated when gene promoters were enriched with TTATCC motif repeat [33]. For this purpose, Tandem Repeat Finder (TRF) [34] was developed to identify tandem repeats. PlantPAN annotates not only transcription factor binding sites, but also CpG/CpNpG islands and tandem repeats in plant promoter sequences, to analyze all of these regulatory features simultaneously. Additionally, as the availability of data from multiple eukaryotic genome sequencing projects increases, attention has been focused on comparative genomic approaches. For that reason, PlantPAN also provides an additional special "Cross-Species" analyzing function for discovering the transcription factor binding sites in conserved regions between promoters of homologous genes or two input sequences. Thus, PlantPAN provides an effective resource for versatile analyses and predictions of the transcriptional regulation of genes in plants.

## Construction and content

**Figure 1:**
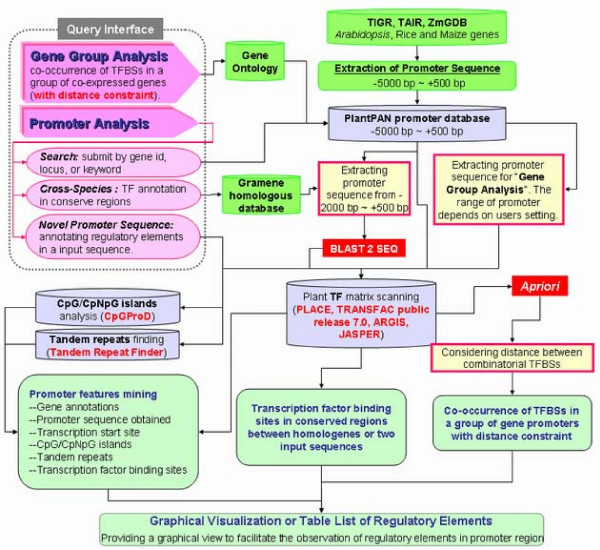
**System flow of PlantPAN**. PlantPAN has two query interfaces. "Gene group analysis" discovers the co-occurrence of TFBSs in a group of gene promoters; "Promoter analysys" contains three subfunctions: "*Search*" and "*Novel promoter sequence*" search TFBSs, CpG/CpNpG islands and tandem repeats in a single input gene ID or a novel input promoter sequence; "*Cross-Species*" identifies TFBSs in conserved regions between homologous or two promoters.

PlantPAN is a web-based system which is running on an Apache web server on a Linux operation system. The content of the integrated databases including gene information, gene ontology (GO), gene sequence, promoter sequence, transcription factor binding sites, CpNpG islands and tandem repeat regions are stored in a MySQL relational database system, and all tables are connected by means of Gene ID (Fig. S1 in additional file 1). All web pages and data parsers are written in PHP and Perl. Figure 1 displays the system flow chart of PlantPAN which lets users query by gene ID, locus, keyword and sequence, and the promoter analysis system. After promoter extraction, the user can efficiently identify the *cis*-regulatory elements within the conserved regions of homologous genes. Moreover, the combinatorial transcription factor binding sites with distance constraint can be identified in a group of gene promoter sequences. The detailed methods are illustrated as follows.

### Integrating external databases

Gene information (gene ID, gene locus, gene description, gene location, GO terms, and genomic sequence) of *Arabidopsis *(*A. thaliana*), *Oryza *(*O. sativa*) and maize (*Z. mays*) was obtained from TAIR (TAIR6_genome_release) [35], TIGR (o_sativa_version_4.0) [36] and ZmGDB [37], respectively. The sequences from 5000 bp upstream to 500 bp downstream of the transcription start site (TSS) (+1) were extracted and defined as the promoter regions of genes in PlantPAN (-2000 bp to +1 bp in maize). In case of genes lacking positional information on the TSS, the translational start site (ATG) was used as point of reference. The annotated information on the homologous genes was obtained from Gramene [38]. The numbers of collected gene transcripts from *Arabidopsis*, *Oryza*, and *Zea *are 35,351, 62,827 and 29,759, respectively. Users are allowed to input the gene IDs [39], locus names or keywords to extract the gene upstream of the input gene or the conserved upstream regions across different species. The transcription factor binding profiles were collected from PLACE, TRANSFAC (public release 7.0), AGRIS and JASPER. Table 1 shows the data statistics of PlantPAN in detail.

**Table 1:** Data statistics of PlantPAN.

	**Arabidopsis**	**Rice**	**Maize**	**Other plants**
No. of gene transcripts	35,351	62,827	29,759	-

No. of promoter sequences	35,351	62,827	29,759	-

No. of experimental promoter sequences	13	6	16	167^a^

No. of transcripts containing putative CpG/CpNpG Islands (predicted by CpGProD)	6,912	60,470	16,110	-

No. of transcripts containing putative tandem repeats (predicted by TRF)	18,080	45,409	11,535	-

No. of plant transcription factors used in PlantPAN^b^	197	75	59	260^c^

^a ^Including medicago, barley, *Catharanthus roseus*, cider tree, french bean, snapdragon, horseradish, maize, parsley, pea, petunia, *Brassica napus*, soybean, potato, tobacco, tomato, and wheat.

^b ^Collecting 591 plant transcription factors from PLACE [16], TRANSFAC public release 7.0 [12], AGRIS [17], and JASPER [18].

^c ^Including bean, barley, carrot, cotton, medicago, parsley, pea, petunia, potato, rape, snapdragon, soybean, sweet potato, tobacco, tomato, wheat et al.

### Identifying cis-regulatory elements

After the promoter region had been determined, the regulatory elements, such as transcription factor binding sites (TFBSs), CpG/CpNpG islands, and tandem repeats were annotated. Table 2 presents numerous methods that were integrated into the system for analyzing the regulatory elements in promoter sequences and input sequences. For example, MATCH [40] detects the transcription factor binding sites in a promoter sequence using the transcription factor binding profiles from TRANSFAC public release 7.0 [12]. The default values of core similarity and matrix similarity of MATCH program were set to 1.0 and 0.75, respectively. Consensus sequence from PLACE [16], AGRIS [17] and JASPER [19] were also used to scan TFBSs in a promoter sequence. Moreover, cytosine DNA methylation in plants is found primarily in transposable elements, CpG/CpNpG islands and repetitive DNA sequences [41,42]. The CpG/CpNpG islands are defined as that DNA regions that are longer than 500 nucleotides, with a moving average C+C frequency of above 0.5 and a moving average CpG/CpNpG observed/expected (o/e) ratio more than 0.6 [29]. CpGProD [29], which searches among all CpG/CpNpG islands located in the query sequences, was integrated into PlantPAN for the detection of CpG/CpNpG islands in promoters. Repeat sequences in gene promoters are important in regulating gene expression. Tandem repeat finder [34], which runs without any specific pattern or pattern size, was applied with minor modifications to find repeat regions in promoters.

**Table 2:** Supported regulatory features in PlantPAN.

**Transcriptional Regulatory Features**	**Integrated Databases or Tools**	**Descriptions**
Promoter sequences and location sites	TAIR [35]	Containing the information on the TSS and sequence location sites of *Arabidopsis *genes from the annotations in TAIR.
	
	TIGR [36]	Containing the information on the TSS and sequence location sites of *Oryza *genes from the annotations in TIGR.
	
	ZmGDB [37]	Containing the information on the 2 kb upstream location sites of *Zea *genes from the annotations in ZmGDB.

Transcription factor binding sites	TRANSFAC public release 7.0 [11-13]	Collecting experimentally verified transcription factors, their genomic binding sites and DNA-binding profiles.
	
	PLACE [16]	A database of nucleotide sequence motifs found in plant *cis*-acting regulatory DNA elements. Motifs were extracted from previously published reports on genes in vascular plants.
	
	AGRIS [17]	Collecting approximately 1,770 *Arabidopsis *transcription factors that are grouped into 50 families.
	
	JASPER [18,19]	A popular open-access database for matrix models describing DNA-binding preferences for transcription factors and other DNA patterns.
	
	MATCH [40]	Scanning transcription factor binding sites using transcription factor binding profiles from TRANSFAC and PLACE.

CpG/CpNpG islands	CpGProD [29]	Detecting CpG/CpNpG islands.

Tandem repeats	TRF [34]	Finding the tandem repeat.

Conservation of homologous gene promoter sequences	BLAST [46]	Searching sequence similarity; it is also used for discovering similar gene promoters and identifying conserved regions in the PlantPAN assistant promoter database.
	
	BL2SEQ [47]	Utilizing the BLAST algorithm for identifying conserved regions in two sequences.

Co-occurrence transcription factor binding sites in a gene group of gene promoters	*Apriori *[43,44]	Mining the co-occurrence of transcription factor binding sites in a group of gene promoters.

### Identifying co-occurrence of TFBSs in a group of gene promoters

**Figure 2:**
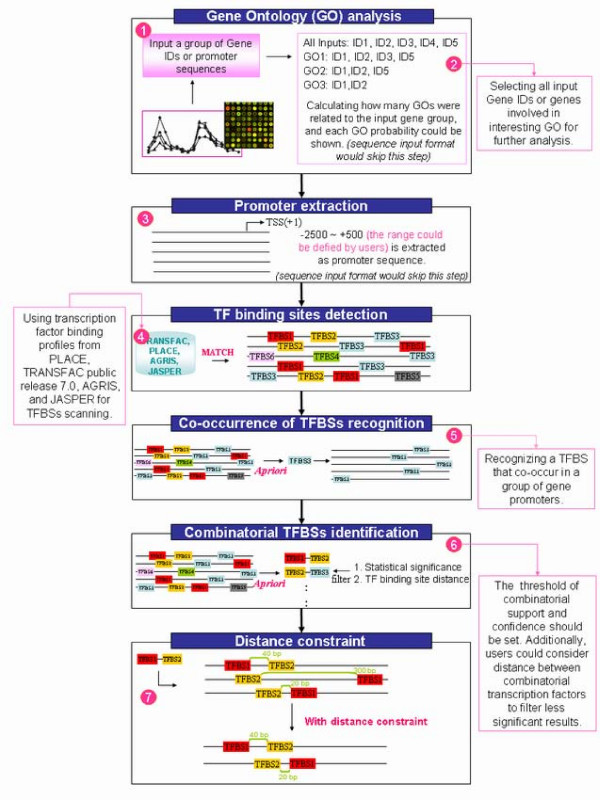
**Gene group analysis in PlantPAN**. The "Gene group analysis" process has seven steps. Following GO function analysis, promoter extraction and TFBS scanning, the co-occurrence of TFBSs and combinatorial TFBSs in a group of gene promoters is tabulated and presented in two figures (with and without distance constraint).

The "Gene group analysis" function of PlantPAN system, which comprises seven analytic steps (Fig. 2), is utilized to discover the co-occurrence of transcription factor binding sites in a group of gene promoters. In the first step, a group of input gene IDs of chosen species (such as AGI for *Arabidopsis *or locus name for *Oryza*) or a group of promoter sequences is allowed for input to the system. In the second step, the system calculates the GO terms related to the input genes. The genes involved in different GO terms are tabulated. Users can choose all genes or genes in a particular GO term for further analysis. In the third step, the promoter sequence is extracted from the PlantPAN promoter database. However, if users input a group of promoter sequences in step one, then the system will skip steps two and three. In the fourth step, users can select transcription factors binding profiles from different species and scan TFBSs in the promoter regions. The thresholds of the core similarity and the matrix similarity should be set in this step; the default values are 1.0 and 0.75, respectively.

In step five, a figure depicts all detected TFBSs in every promoter. Consequently, *Apriori *is a program that is implemented to mine association rules for a group of input data [43,44]. A set of transcription factors, which bind to target sites, is believed to participate in regulating gene transcription [44]. In this study, *Apriori *was used to discover the co-occurrence of transcription factor binding sites (TFBSs) and combinatorial TFBSs in a group of gene promoters (Fig. S2 in additional file 1). An important parameter, namely *Support*, is the probability that the promoters *D *contain a TFBS *A *or the combinatorial TFBSs *A *and *B*. After the co-occurrences of TFBSs in the group of gene promoter sequences have been mined, the statistical significance of each TFBS should be examined against the background set of gene promoters, based on the hypergeometric equation (*p*-value) [4].

$$\text{P}(\text{t})=\sum_{\text{t}}^{\text{T}}\frac{{\text{C}}_{\text{t}}^{\text{T}}\times {\text{C}}_{\text{k}-\text{t}}^{\text{K}-\text{T}}}{{\text{C}}_{\text{k}}^{\text{K}}}$$

where *K *is the number of background gene promoters used and *T *is the number of observed gene promoters that are input by users, *k *is the number of promoters have the combination in the background gene set and *t *is the number of promoters have the combination in the observed gene set. *P*-value is calculated for each combination based on the hypermetric equation; smaller the *p*-value is, more statistically significant the combination is. A smaller *p*-value of a combination corresponds to greater statistical significance.

One TFBS which co-occur in a group of gene promoters could be identified in sixth step. Additionally, the fact that target genes with characteristic distances show significantly higher co-expression than those without preferred distances provides evidence for the biological relevance of the observed characteristic distances [45]. Yu *et al*. found that 75% of the interacting transcription factors were occurred within the characteristic distances which are smaller than 166 bp in yeast [45]. In this work, a distance of 20 to 200 bp between two factors is considered to analyze the co-occurrence of combinatorial TFBSs in gene group. Accordingly, the support and confidence values in co-occurrence analysis and a distance constraint must be set in step six. Following the six-step analysis, step seven (final step) displays the co-occurrence percentage of every pair of combinatorial TFBSs for the input genes. Finally, users can investigate the interested combinations of TFBSs within the defined distance by graphical laid-out.

### Identifying TFBSs, tandem repeats, and CpNpG islands in homologous conserved regions

The paralogous and orthologous genes among *Arabidopsis *and *Oryza *in the cross-species analysis of promoter sequences of homologous genes, were extracted from Gramene [38]. Following the identification of the paired homologous genes, the sequence alignment search tool, BLAST [46], was applied to identify conserved regions in promoter sequences. Based on the conservation of homologous promoter sequences, transcription factor binding sites within the conserved regions are identified. Users can input a promoter sequence to search for homologous gene promoters; this capacity diversifies the platform. Additionally, two sequences in FASTA format can be employed to search for conserved regions within the two sequences using BL2SEQ [47] program. The detection of transcription factor binding sites, tandem repeats, and CpNpG islands in those regions are also displayed. The identified conserved sites are more believable than those non-conserved regions in the analyses of the transcriptional regulation in plant genes.

### Graphical visualization and table list

The regulatory features discovered in the promoters are presented graphically or tabulated. A graphical interface is implemented using the GD library of a PHP programming language. Once the analysis has been completed, numerous regulatory characteristics, including transcription factor binding sites, CpG/CpNpG islands, and repeat regions, are shown in an overview. The regulatory features are then presented in more detail if users click the regulatory elements figured in the graph or the label, "View in Table." Moreover, the regulatory elements in the conserved regions and the co-occurrence of *cis*-regulatory elements are also revealed graphically to improve presentation.

## Utility and discussion

**Figure 3:**
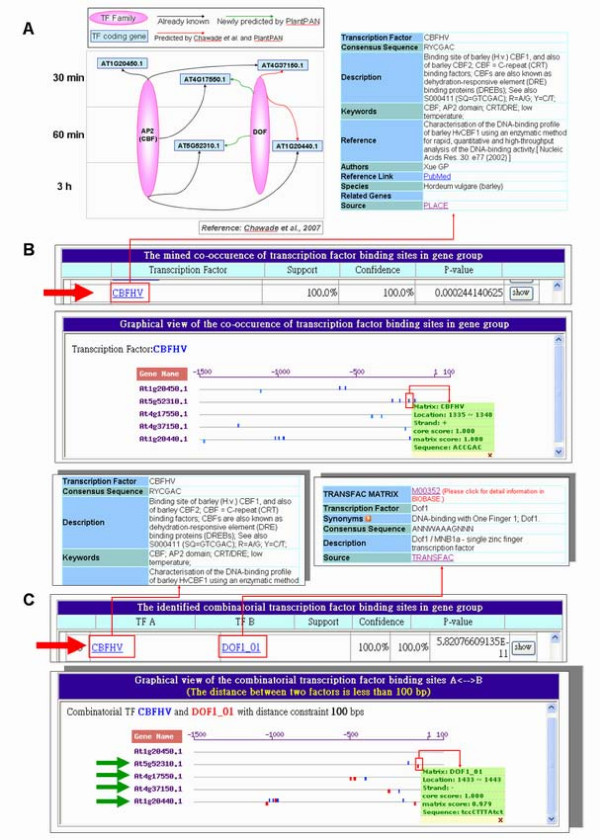
**Results of case study I in "Gene group analysis"**. (A) Reference case taken from Chawade *et al*., 2007 [10]. The genes used in the case study are At4g17550.1, At1g20450.1, At5g52310.1, At4g37150.1, and At1g20440.1. The origin of the arrow indicates the regulating TF family and the endpoint of the arrow indicates the target gene. The time scale shown on the vertical axis is cold treatment of plant. (B) CBFHV (AP2) displayed co-occurrences in At4g17550.1, At1g20450.1, At5g52310.1, At4g37150.1, and At1g20440.1 (C) CBFHV (AP2) and DOF represented combinatorial co-occurrences in At5g52310.1, At4g17550.1, At4g37150.1, and At1g20440.1 with 100 bp distance constraint between CBFHV and DOF.

PlantPAN has two main functions. Firstly, it applies "Gene group analysis" to identify the co-occurrence of transcription factor binding sites in a group of gene promoters. Combinatorial regulation by transcription factor complexes is an important characteristic of eukaryotic gene regulation [3,4,45]. Two case studies are performed to elucidate the biological utility of "Gene group analysis" (Fig. 3 and S3 in additional file 1). Secondly, it applies "Promoter analysis" to analyze the TFBSs, CpG/CpNpG islands and tandem repeats in the promoter sequence of a given gene ID or a novel promoter sequence. The homologene of an input gene ID can be extracted, and the TFBSs in the conserved regions between two promoter sequences identified. However, one or two input promoter sequences are allowed. Default options have been set for all tools that yield easily understandable results, and all of the graphical results can be clicked for further explanation.

### Gene group analysis – case study I

In a previous study, Chawade *et al*. [10] constructed putative cold regulatory networks by integrating data from co-expressed microarray data, promoter sequences and known promoter binding sites. In a part of this regulatory network, co-expressed cold related genes, At4g17550.1, At1g20450.1, At5g52310.1, At4g37150.1, and At1g20440.1 were all regulated by AP2 following cold treatment for 30 min in microarray data (Fig. 3A). These five gene IDs were used as inputs in the "Gene group analysis" of PlantPAN. Transcription factors from all plant species were chosen to detect TFBSs in promoters. The thresholds of the core and matrix scores in TFBSs scanning and the support and confidence values in the co-occurrence analysis were all set to their default values. In this example, a distance of 100 bp between two factors was used to analyze the co-occurrence of combinatorial TFBSs. Consequently, the six analytic steps identified CBFHV (AP2) in these five promoters (Fig. 3B). This result was confirmed an already known regulatory pathway, as described earlier [10]. Moreover, Chawade *et al*. predicted that DOF and AP2 could co-regulate At4g37150.1 and At1g20440.1 in this cold regulatory network [10] (Fig. 3A). Significantly, DOF and AP2 were also identified as combinatorial transcription factors in At4g37150.1 and At1g20440.1 promoters after seven-step analysis in the PlantPAN system (Figs. 3A and 3C). Two pathways were newly predicted: DOF may regulate AT5G52310.1 and At4G17550.1 expression and co-occur with AP2 in a cold regulatory network (Figs. 3A and 3C). Accordingly, this system can be adopted to analyze co-regulation in microarray gene expression databases, such as AtGenExpress [48] and Genevestigator [49]. The developed PlantPAN system improves our understanding of the transcription regulatory networks of gene regulation in plants.

### Gene group analysis – case study II

The development of flowers has attracted widespread interest in recent decades as an excellent model system of plant development. A novel floral induction system was recently used to construct an early *Arabidopsis *flower development network [50]. Particular transcription factors regulated various co-expressed genes, demonstrating the critical roles of such genes in flower development [50]. Some genes in this gene regulation network are taken as an example to demonstrate the effectiveness of the developed "Gene group analysis" system. Wellmer *et al*. indicated that AP1 regulated TFL1 (At5g03840.1), LFY (At5g61850.1), FUL (At5g60910.1), AGL24 (At4g24540.1), and PI (At5g20240.1), which participated importantly in flower development (Fig. S3A in additional file 1) [50]. These five gene IDs were input into the "Gene group analysis". Again, transcription factors from all plant species were selected to detect TFBSs in promoters. The thresholds of the core and matrix scores in TFBSs scanning and the support and confidence values in co-occurrence analysis were set to the default values. In this case study, a distance of 100 bp between two factors is considered to analyze the co-occurring TFBSs. Consequently, the six analytic steps identified AP1 in these five promoters (Fig. S3B in additional file 1). This result was confirmed using Wellmer's model [50]. However, the most remarkable utility of the proposed system is not its identification of a single transcription factor that may regulate a group of genes, but the identification of candidates that may co-occur with the finding TF. This information yields the novel transcription factor binding sites or supports the discovery of co-regulated transcription factors. Furthermore, the distance between the two co-occurring transcription factors was regarded as important in regulating transcription. In this example, the C1-motif (CIMOTIFZMBZ2) might co-occur with AP1 in the group of genes within a distance of less than 100 bp (Fig. S3C in additional file 1). The C1-motif has also been demonstrated to be required for anthocyanin pigmentation in the aleuron and scutellum of the plant biological kernels [51,52]. As a result, the C1-motif might be a new candidate that is involved in the regulation of flower development in plants and might be co-regulated with AP1. Therefore, this system can be utilized to identify novel TFBSs.

### Promoter analysis – annotating TFBSs, CpG/CpNpG islands, andtandem repeats

**Figure 4:**
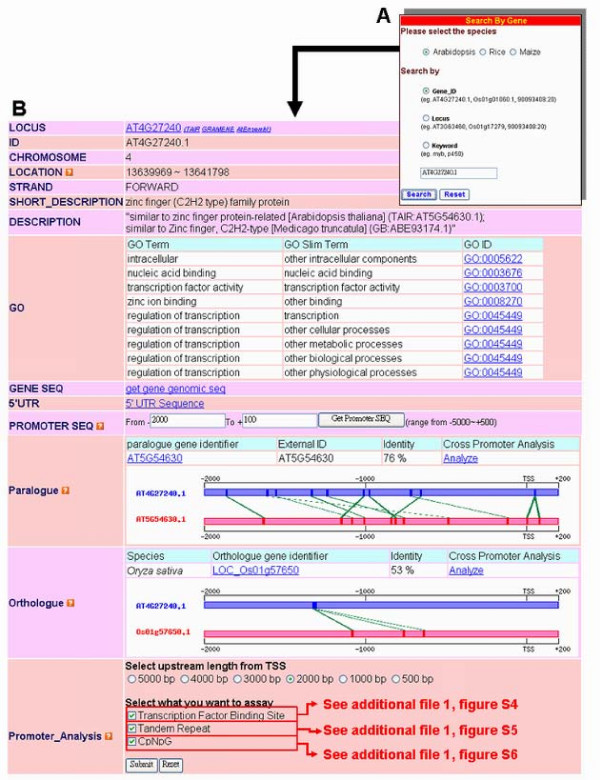
**Web interface for a search for a single gene in PlantPAN**. The "Search" web tool can be used to search for general gene information and gene regulatory features; furthermore, (B) tabulated results contain general gene information and "Promoter Analysis" functions. The "Promoter analysis" functions can be used to identify regulatory elements in the promoter sequence.

Figure 4 depicts the "Search" interface of the PlantPAN. Users should select a species of interest (*Arabidopsis*, or rice, or maize) (Fig. 4A), and then the input gene ID, the locus name, or keywords to identify general gene annotations (chromosome, location, strand, gene description, GO, gene sequence, promoter sequence, 5' UTR sequence, paralogene, and orthologene). Following system analysis, the results of a single gene search are tabulated. A "Promoter analysis" function at the bottom of the table can be employed to find various regulatory elements in the gene promoter (Fig. 4B). Several case studies of *Arabidopsis *described below, demonstrate the proposed system.

In the annotation of TFBSs, *Arabidopsis thaliana *rbcS-1A (At1g67090.1) promoter has been defined from -320 bp to -125 bp; a binding site (CTTCCACGTGGCA, from -241 bp to -230 bp) is present for the GBF (G-box binding factor) transcription factor binding[53]. Following the input of the *Arabidopsis *rbcS-1A gene ID for a search, one GBF binding site was identified between -241 bp and -230 bp (Fig. S4 in additional file 1). The graph is hyperlinked to more details of the transcription factor or TFBSs.

Previous investigations have revealed that the gene expression can be up-regulated when the promoter that contains Up1 (GGCCCAWW) or Up2 (AAACCCTA) repeats [33]. *Arabidopsis *nucleolar protein (AT4G26600.1) is one of the putative genes whose promoter contains Up1 and Up2 [33]. These repeats were successfully identified by PlantPAN in the At4G26600.1 promoter (Fig. S5 in additional file 1). In the annotation of CpG/CpNpG islands, several methyl-CpG-binding domain (MBD) proteins [54], which contain CpG/CpNpG islands, were identified; PlantPAN exhibits those at -2342 bp to -1480 bp in the MBD5 (AT3G46580.1) promoter region (Fig. S6 in additional file 1).

Nevertheless, users can input a novel promoter sequence to analyze the above four regulatory features. After the annotation tools were employed, the selected features, such as TFBSs, CpG/CpNpG islands and tandem repeats, were represented in the graph and table (Figs. S4-S6 in additional file 1). The parameters of each annotating tool were set to their default values, as described in Construction and content.

### Cross-Species

**Figure 5:**
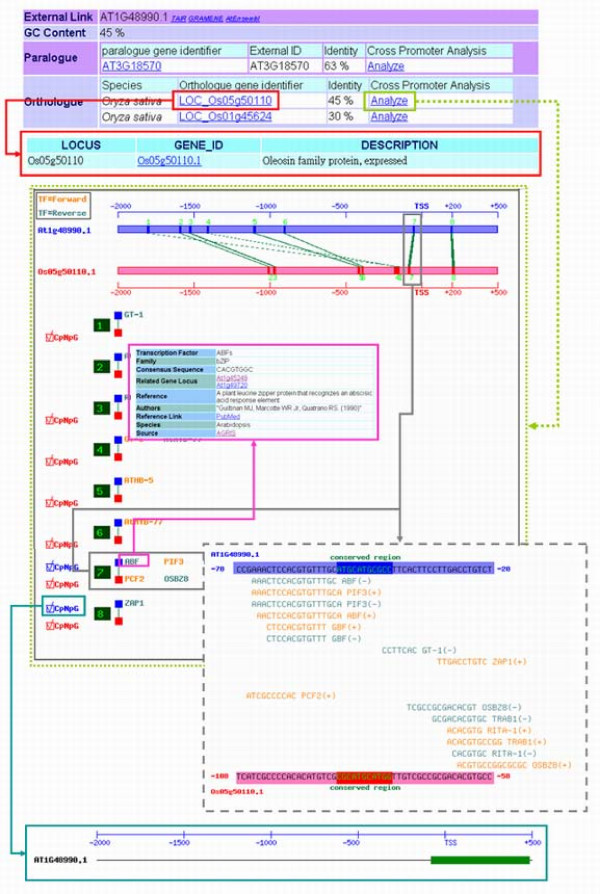
**Graphical view of a case (AT1G48990) of "Cross-Species" analysis**. The conserved regions and TFBSs in the conserved regions are shown in a figure significantly. Each conserved site or TFBS can be further clicked for more detailed information.

"Cross-Species" is one of the three subfunctions in "Promoter analysis". It identifies the transcription factor binding sites, CpG/CpNpG islands, and tandem repeats in the conserved regions of the promoters in paralogous or orthologous genes. The proposed system can conveniently perform an analysis by the direct input of the gene accession in the selected species, a single promoter sequence or two sequences in FASTA format. After the input data are processed, the paired sequences are displayed in distinct colors to distinguish the conserved regions from the non-conserved regions. The sequences of regulatory sites are implied (Fig. 5). For instance, previous studies have established that ABI3 binding to the upstream sequence of oleosin in *Arabidopsis *regulates oleosin gene expression [55]. However, no experiment on the gene regulation of *Oryza *oleosin has been reported upon. "Cross-Species" analysis in PlantPAN indicates many transcription factor binding sites (including ABF, which is an ABA response binding factor), as predicted in the conserved regions between -58 bp and -48 bp and between -78 bp and -88 bp in *Arabidopsis *(AT1G48990) and *Oryza *(LOC_Os05g50110), respectively (Fig. 5). These results open up a new avenue for further studies of oleosin in *Oryza*. Comparative genomic approaches are having a remarkable effect on the study of transcriptional regulation in eukaryotes. Therefore, the conserved regions may be candidate regulatory modules for further experimentation.

### Future development

The number of sequenced and annotated plant genomes is rapidly increasing. The PlantPAN database is currently being expanded to cover species other than *Arabidopsis*, rice and maize. Future versions will include other plant species (wheat, potato, barley and others). Additionally, the transcription factors will be enlarged by taking into account more experimental matrices from different plants. The authors will in the near future be energetically connecting transcription factors to other proteins using protein-protein interaction databases. Furthermore, the plant microarray data will be integrated into "Gene group analysis" of PlantPAN.

## Conclusion

PlantPAN provides a "Gene group analysis" function for analyzing the co-occurrence of combinatorial TFBSs with a distance constraint in sets of plant genes. This function extends a good platform to examine the co-expression genes of microarray data in transcriptional regulation networks. Furthermore, the PlantPAN web server not only provides a user-friendly input/output interface, but also offers numerous advantages in plant promoter analysis over currently available tools for annotating plant promoters (Table S1 in additional file 1). PlantPAN supports various important regulatory elements for promoter analysis, such as transcription factor binding sites, CpG/CpNpG islands, and tandem repeat regions. PlantPAN also provides "Cross-Species" analysis for two paralogous or orthologous promoters, allowing the identification of transcription factor binding sites to be refined. Future improved versions of PlantPAN will include more detailed information on gene regulation and transcription factors. The PlantPAN resource will be continuously maintained and updated for upcoming studies.

## Availability and requirements

Access to PlantPAN is via a web interface, freely available to all interested users, at .

## List of abbreviations

GO: gene ontology; TFs: transcription factors; TFBSs: transcription factor binding sites; TSS: transcription start site.

## Authors' contributions

HDH and RLP conceived and supervised the project. WCC was responsible for the design, computational analyses, implemented the databases, web interface development and draft the manuscript with revisions provided by HDH and RLP. TYL participated in the design, computational analyses, web interface development and systems maintained. HYH helped with web interface development, system maintained, and data testing. All authors read and approved the final manuscript.

## Supplementary Material

Additional File 1 **Supplementary figures (S1, S2, S3, S4, S5 and S6) and table (S1)**. The data provided represent six supplementary figures and one supplementary table in this study. Click here for file

## References

[B1] Gong W, Shen YP, Ma LG, Pan Y, Du YL, Wang DH, Yang JY, Hu LD, Liu XF, Dong CX (2004). Genome-wide ORFeome cloning and analysis of *Arabidopsis *transcription factor genes. Plant Physiol.

[B2] Wray GA, Hahn MW, Abouheif E, Balhoff JP, Pizer M, Rockman MV, Romano LA (2003). The evolution of transcriptional regulation in eukaryotes. Mol Biol Evol.

[B3] Balaji S, Babu MM, Iyer LM, Luscombe NM, Aravind L (2006). Comprehensive analysis of combinatorial regulation using the transcriptional regulatory network of yeast. J Mol Biol.

[B4] Kato M, Hata N, Banerjee N, Futcher B, Zhang MQ (2004). Identifying combinatorial regulation of transcription factors and binding motifs. Genome Biol.

[B5] Galuschka C, Schindler M, Bulow L, Hehl R (2007). AthaMap web tools for the analysis and identification of co-regulated genes. Nucleic Acids Res.

[B6] Steffens NO, Galuschka C, Schindler M, Bulow L, Hehl R (2005). AthaMap web tools for database-assisted identification of combinatorial *cis*-regulatory elements and the display of highly conserved transcription factor binding sites in *Arabidopsis thaliana*. Nucleic Acids Res.

[B7] Bulow L, Schindler M, Hehl R (2007). PathoPlant: a platform for microarray expression data to analyze co-regulated genes involved in plant defense responses. Nucleic Acids Res.

[B8] Jen C-H, Manfield IW, Michalopoulos I, Pinney JW, Willats WGT, Gilmartin PM, Westhead DR (2006). The *Arabidopsis *co-expression tool (act): a WWW-based tool and database for microarray-based gene expression analysis. The Plant Journal.

[B9] Obayashi T, Kinoshita K, Nakai K, Shibaoka M, Hayashi S, Saeki M, Shibata D, Saito K, Ohta H (2007). ATTED-II: a database of co-expressed genes and cis elements for identifying co-regulated gene groups in Arabidopsis. Nucleic Acids Res.

[B10] Chawade A, Brautigam M, Lindlof A, Olsson O, Olsson B (2007). Putative cold acclimation pathways in *Arabidopsis thaliana *identified by a combined analysis of mRNA co-expression patterns, promoter motifs and transcription factors. BMC Genomics.

[B11] Wingender E, Chen X, Hehl R, Karas H, Liebich I, Matys V, Meinhardt T, Pruss M, Reuter I, Schacherer F (2000). TRANSFAC: an integrated system for gene expression regulation. Nucleic Acids Res.

[B12] Wingender E, Karas H, Knuppel R (1997). TRANSFAC database as a bridge between sequence data libraries and biological function. Pac Symp Biocomput.

[B13] Matys V, Kel-Margoulis OV, Fricke E, Liebich I, Land S, Barre-Dirrie A, Reuter I, Chekmenev D, Krull M, Hornischer K (2006). TRANSFAC and its module TRANSCompel: transcriptional gene regulation in eukaryotes. Nucleic Acids Res.

[B14] O'Connor TR, Dyreson C, Wyrick JJ (2005). Athena: a resource for rapid visualization and systematic analysis of Arabidopsis promoter sequences. Bioinformatics.

[B15] Riano-Pachon DM, Ruzicic S, Dreyer I, Mueller-Roeber B (2007). PlnTFDB: an integrative plant transcription factor database. BMC Bioinformatics.

[B16] Higo K, Ugawa Y, Iwamoto M, Korenaga T (1999). Plant *cis*-acting regulatory DNA elements (PLACE) database: 1999. Nucleic Acids Res.

[B17] Davuluri RV, Sun H, Palaniswamy SK, Matthews N, Molina C, Kurtz M, Grotewold E (2003). AGRIS: Arabidopsis gene regulatory information server, an information resource of Arabidopsis cis-regulatory elements and transcription factors. BMC Bioinformatics.

[B18] Bryne JC, Valen E, Tang MH, Marstrand T, Winther O, da Piedade I, Krogh A, Lenhard B, Sandelin A (2008). JASPAR, the open access database of transcription factor-binding profiles: new content and tools in the 2008 update. Nucleic Acids Res.

[B19] Sandelin A, Alkema W, Engstrom P, Wasserman WW, Lenhard B (2004). JASPAR: an open-access database for eukaryotic transcription factor binding profiles. Nucleic Acids Res.

[B20] Guo A, He K, Liu D, Bai S, Gu X, Wei L, Luo J (2005). DATF: a database of *Arabidopsis *transcription factors. Bioinformatics.

[B21] Lescot M, Dehais P, Thijs G, Marchal K, Moreau Y, Peer Y Van de, Rouze P, Rombauts S (2002). PlantCARE, a database of plant cis-acting regulatory elements and a portal to tools for in silico analysis of promoter sequences. Nucleic Acids Res.

[B22] Jeddeloh JA, Bender J, Richards EJ (1998). The DNA methylation locus DDM1 is required for maintenance of gene silencing in *Arabidopsis*. Genes Dev.

[B23] Rombauts S, Florquin K, Lescot M, Marchal K, Rouze P, Peer Y van de (2003). Computational approaches to identify promoters and cis-regulatory elements in plant genomes. Plant Physiol.

[B24] Kooter JM, Matzke MA, Meyer P (1999). Listening to the silent genes: transgene silencing, gene regulation and pathogen control. Trends Plant Sci.

[B25] Vaucheret H, Fagard M (2001). Transcriptional gene silencing in plants: targets, inducers and regulators. Trends Genet.

[B26] Pradhan S, Urwin NA, Jenkins GI, Adams RL (1999). Effect of CWG methylation on expression of plant genes. Biochem J.

[B27] Cao X, Jacobsen SE (2002). Locus-specific control of asymmetric and CpNpG methylation by the DRM and CMT3 methyltransferase genes. Proc Natl Acad Sci USA.

[B28] Lindroth AM, Cao X, Jackson JP, Zilberman D, McCallum CM, Henikoff S, Jacobsen SE (2001). Requirement of CHROMOMETHYLASE3 for maintenance of CpXpG methylation. Science.

[B29] Ponger L, Mouchiroud D (2002). CpGProD: identifying CpG islands associated with transcription start sites in large genomic mammalian sequences. Bioinformatics.

[B30] Takai D, Jones PA (2003). The CpG island searcher: a new WWW resource. In Silico Biol.

[B31] Ludwig DL, Chen F, Peterson SR, Nussenzweig A, Li GC, Chen DJ (1997). Ku80 gene expression is Sp1-dependent and sensitive to CpG methylation within a novel cis element. Gene.

[B32] Rasmussen TB, Donaldson IA (2006). Investigation of the endosperm-specific sucrose synthase promoter from rice using transient expression of reporter genes in guar seed tissue. Plant Cell Rep.

[B33] Tatematsu K, Ward S, Leyser O, Kamiya Y, Nambara E (2005). Identification of *cis*-elements that regulate gene expression during initiation of axillary bud outgrowth in *Arabidopsis*. Plant Physiol.

[B34] Benson G (1999). Tandem repeats finder: a program to analyze DNA sequences. Nucleic Acids Res.

[B35] Rhee SY, Beavis W, Berardini TZ, Chen G, Dixon D, Doyle A, Garcia-Hernandez M, Huala E, Lander G, Montoya M (2003). The *Arabidopsis *Information Resource (TAIR): a model organism database providing a centralized, curated gateway to *Arabidopsis *biology, research materials and community. Nucleic Acids Res.

[B36] Yuan Q, Ouyang S, Wang A, Zhu W, Maiti R, Lin H, Hamilton J, Haas B, Sultana R, Cheung F (2005). The institute for genomic research Osa1 rice genome annotation database. Plant Physiol.

[B37] A *Zea mays *Plant Genome Database (ZmGDB). http://www.plantgdb.org/ZmGDB/index.php.

[B38] Jaiswal P, Ni J, Yap I, Ware D, Spooner W, Youens-Clark K, Ren L, Liang C, Zhao W, Ratnapu K (2006). Gramene: a bird's eye view of cereal genomes. Nucleic Acids Res.

[B39] Yanagisawa S (2004). Dof domain proteins: plant-specific transcription factors associated with diverse phenomena unique to plants. Plant Cell Physiol.

[B40] Kel AE, Gossling E, Reuter I, Cheremushkin E, Kel-Margoulis OV, Wingender E (2003). MATCH: A tool for searching transcription factor binding sites in DNA sequences. Nucleic Acids Res.

[B41] Tran RK, Henikoff JG, Zilberman D, Ditt RF, Jacobsen SE, Henikoff S (2005). DNA methylation profiling identifies CG methylation clusters in *Arabidopsis *genes. Curr Biol.

[B42] Bender J (2004). DNA methylation and epigenetics. Annu Rev Plant Biol.

[B43] Srikant R, Vu Q, Agrawal R (1995). Mining generalized association rules. Proceedings of 21st International Conference on Very Large Databases.

[B44] Huang HD, Horng JT, Chang CH, Tsou TS, Hong JY, Liu BJ (2004). A computational approach to discover differential cooperation of regulatory sites in functionally related genes in yeast genome. Journal of Information Science and Engineering.

[B45] Yu X, Lin J, Masuda T, Esumi N, Zack DJ, Qian J (2006). Genome-wide prediction and characterization of interactions between transcription factors in *Saccharomyces cerevisiae*. Nucleic Acids Res.

[B46] McGinnis S, Madden TL (2004). BLAST: at the core of a powerful and diverse set of sequence analysis tools. Nucleic Acids Res.

[B47] Tatusova TA, Madden TL (1999). BLAST 2 Sequences, a new tool for comparing protein and nucleotide sequences. FEMS Microbiol Lett.

[B48] Kilian J, Whitehead D, Horak J, Wanke D, Weinl S, Batistic O, D'Angelo C, Bornberg-Bauer E, Kudla J, Harter K (2007). The AtGenExpress global stress expression data set: protocols, evaluation and model data analysis of UV-B light, drought and cold stress responses. Plant J.

[B49] Zimmermann P, Hirsch-Hoffmann M, Hennig L, Gruissem W (2004). GENEVESTIGATOR. *Arabidopsis *microarray database and analysis toolbox. Plant Physiol.

[B50] Wellmer F, Alves-Ferreira M, Dubois A, Riechmann JL, Meyerowitz EM (2006). Genome-wide analysis of gene expression during early *Arabidopsis *flower development. PLoS Genet.

[B51] Martin C, Prescott A, Mackay S, Bartlett J, Vrijlandt E (1991). Control of anthocyanin biosynthesis in flowers of *Antirrhinum majus*. Plant J.

[B52] Bodeau JP, Walbot V (1996). Structure and regulation of the maize Bronze2 promoter. Plant Mol Biol.

[B53] Donald RG, Cashmore AR (1990). Mutation of either G box or I box sequences profoundly affects expression from the *Arabidopsis *rbcS-1A promoter. Embo J.

[B54] Zemach A, Grafi G (2007). Methyl-CpG-binding domain proteins in plants: interpreters of DNA methylation. Trends Plant Sci.

[B55] Crowe AJ, Abenes M, Plant A, Moloney MM (2000). The seed-specific transactivator, ABI3, induces oleosin gene expression. Plant Science.

