# Supplementary material for: PlantPAN: Plant promoter analysis navigator, for identifying combinatorial cis-regulatory elements with distance constraint in plant gene groups
Source: BMC Genomics. 2008 Nov 26;9:561. doi: 10.1186/1471-2164-9-561 (PMC2633311; doi:10.1186/1471-2164-9-561)
Supplement: Additional File 1 — Supplementary figures (S1, S2, S3, S4, S5 and S6) and table (S1). The data provided represent six supplementary figures and one supplementary table in this study. [file 1471-2164-9-561-S1.doc]

**Additional file 1**

**Figure S1 - Database schema of PlantPAN.**

Table name appear in red background is a single table structures of a single gene searching in PlantPAN. The tables **GO**, **Gene sequence**, **5’ UTR**, **Promoter sequence**, **Paralogue**, **Orthologue**, **TFBSs**, **CpG/CpNpG islands**, and **Tandem repeats** are connected to the PlantPAN main table (red background) by means of the **Gene ID** field. The main keys appear in green background. The **Cross promoter analysis figure output** (yellow background) is connected to **Paralogue** or **Orthologue** by means of the **Gene ID** field. The significant function of PlantPAN is “**Gene Group Analysis**” (blue background). The output tables are also connected to PlantPAN single gene search database by means of **Gene ID** field.

**Figure S2 - An illustrative example for mining the combinatorial transcription factor binding sites.**

A mining association rules method namely *apriori* (3) is used to mine the co-occurrence of transcription factor binding sites (TFBSs) in a group of gene promoter sequences. Consider a large database with transactions, where each transaction consists of a set of items. An association rule is an expression such as *A* => *B*, where *A* and *B* are the sets of items. The related mining association rule is that a transaction in the database that contains *A* also tends to contain *B*. For example, 90% of the people who purchase beer also purchase diapers. Herein, 90% is called the confidence of the rule. The support of the rule *A* => *B* used here is the percentage of transactions that contain both *A* and *B*. The formal statement of the problem is described below. Let *S* = {*s*1, *s*2, …, *sm*} be a set of known transcription factor binding sites of human in TRANSFAC. The union of the members in the set *S* is called ‘item set’. Let *G* = {*g*1, *g*2, …, *gm*} be a group of genes with differential expression in a specific tissue. Each promoter region of a gene is mapped to a transaction containing a set of known regulatory sites, also called items. Assume that a promoter region *S* contains *A*, a set of items of *I*, if *A* ⊆ *S*. An association rule is an implication of the form *A* => *B*, where *A* ⊂ *I*, *B* ⊂ *I*, and *A* ∩ *B* = φ. The rule *A* => *B* holds in the set of promoter regions *D* with *confidence conf* if *c*% of transactions in *D* contains both *A* and *B*. The rule *A* => *B* has *support sup* in the repetitive sequence set *D* if *s*% of promoter regions in *D* contain *A* ∪ *B*. The association rules, the so-called co-occurrence of TFBSs, are generated if the rule has a higher support and confidence than specified by the user.

**Figure S3 - Results of case study II in “Gene group analysis”.**

(A) Reference case taken from Wellmer *et al*., 2006 [44]. The genes used in the case study are marked in yellow boxes. (B) AP1 displayed co-occurrences in TFL1 (At5g03840.1), LFY (At5g61850.1), FUL (At5g60910.1), AGL24 (At4g24540.1) and PI (At5g20240.1). (C) AP1 and C1-motif (C1MOTIFZMBZ2) represented combinatorial co-occurrences in TFL1 (At5g03840.1), LFY (At5g61850.1), FUL (At5g60910.1), AGL24 (At4g24540.1) and PI (At5g20240.1).

**Figure S4 -** **Identification of transcription factor binding sites in AT1G67090.1.**

**Figure S5 - Identification of tandem repeat regions in upstream sequence of AT4G26600.1.**

**Figure S6 -** **Identification of CpG/CpNpG islands in AT3G46580.1.**

**Table S1 - Comparison of PlantPAN with other plant promoter analysis systems.**


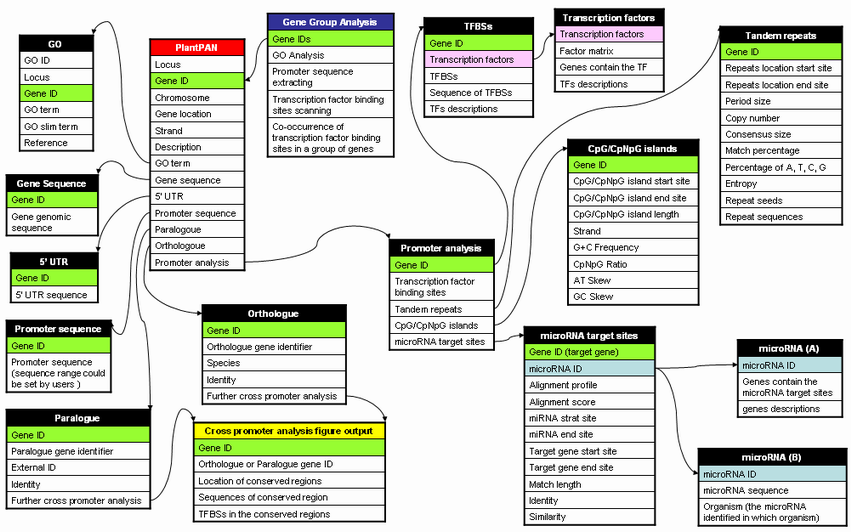


**Figure S1 - Database schema of PlantPAN.**


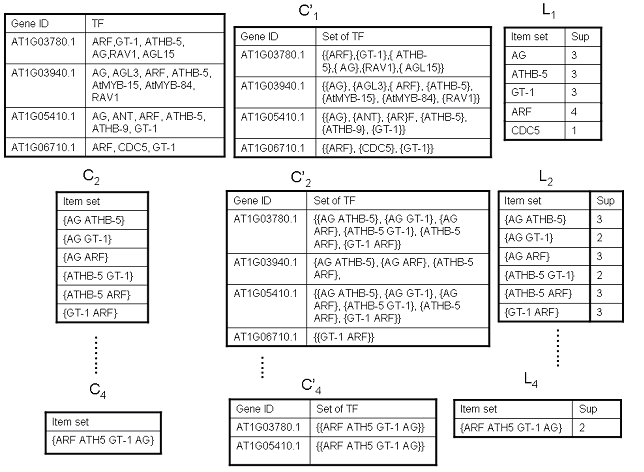


**Figure S2 - An illustrative example for mining the combinatorial transcription factor binding sites.**


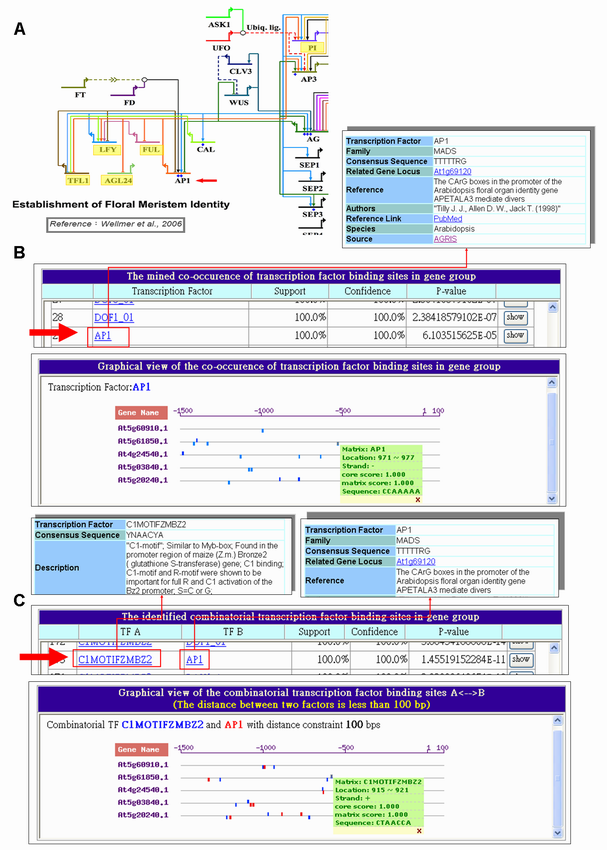


**Figure S3 - Results of case study II in “Gene group analysis”.**

**
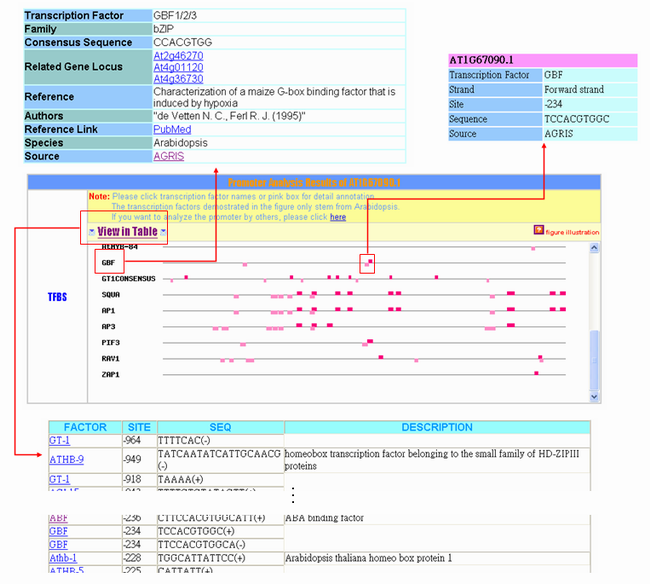
**

**Figure S4 -** **Identification of transcription factor binding sites in AT1G67090.1.**

**
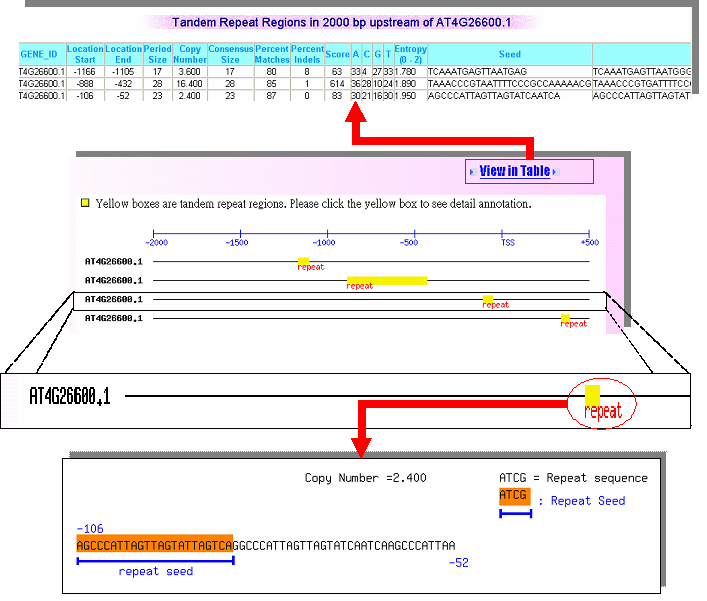
**

**Figure S5 - Identification of tandem repeat regions in upstream sequence of AT4G26600.1.**

**
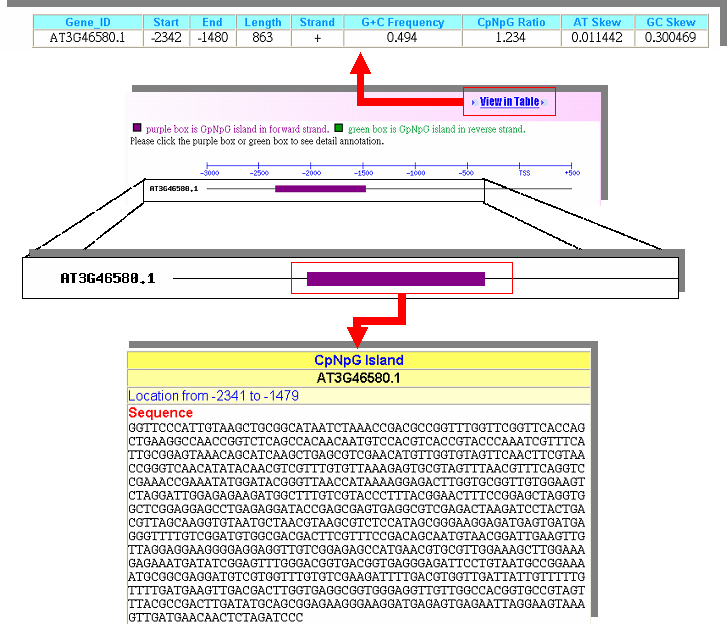
**

**Figure S6 -** **Identification of CpG/CpNpG islands in AT3G46580.1.**

**Table S1 - Comparison of PlantPAN with other plant promoter analysis systems.**

| **Comparing items** | **AGRIS** | **AthaMap** | **PLACE** | **PlantCARE** | **PlantPAN** |
| --- | --- | --- | --- | --- | --- |
| Reference | Davuluri *et al*., 2005 | Steffens *et al*., 2005 | Higo *et al.*, 1999 | Lescot *et al*,. 2002 | **-** |
| Transcription factor binding sites | Yes | Yes | Yes | Yes | **Yes**  (TRANSFAC, PLACE, MATCH) |
| CpG/CpNpG islands | - | - | - | - | **Yes**  (CpGProD) |
| Tandem repeats | - | - | - | - | **Yes**  (Tandem Repeat Finder) |
| Cross-species comparison of homologous gene promoter sequences | - | - | Yes | - | **Yes** |
| Combinatorial co-occurrence of transcription factor binding sites | - | Yes | - | - | **Yes**  (*Aprior,* distance constraint) |
| On-line analysis for regulatory features | - | - | Yes | Yes | **Yes** |
| Species supported | *Arabidopsis* | *Arabidopsis* | plant TFs | plant TFs | **plant TFs.** (***Arabidopsis*, *rice,*** and ***maize***) |
